# Sniff-Like Patterned Input Results in Long-Term Plasticity at the Rat Olfactory Bulb Mitral and Tufted Cell to Granule Cell Synapse

**DOI:** 10.1155/2016/9124986

**Published:** 2016-09-26

**Authors:** Mahua Chatterjee, Fernando Perez de los Cobos Pallares, Alex Loebel, Michael Lukas, Veronica Egger

**Affiliations:** ^1^Department of Biology II, Ludwig-Maximilians-University, Grosshadernerstr. 2a, 82152 Martinsried, Germany; ^2^Regensburg Center of Neuroscience, Regensburg University, Universitätsstr. 30, 93040 Regensburg, Germany; ^3^Bernstein Center for Computational Neuroscience, Grosshadernerstr. 2a, 82152 Martinsried, Germany

## Abstract

During odor sensing the activity of principal neurons of the mammalian olfactory bulb, the mitral and tufted cells (MTCs), occurs in repetitive bursts that are synchronized to respiration, reminiscent of hippocampal theta-gamma coupling. Axonless granule cells (GCs) mediate self- and lateral inhibitory interactions between the excitatory MTCs via reciprocal dendrodendritic synapses. We have explored long-term plasticity at this synapse by using a theta burst stimulation (TBS) protocol and variations thereof. GCs were excited via glomerular stimulation in acute brain slices. We find that TBS induces exclusively long-term depression in the majority of experiments, whereas single bursts (“single-sniff paradigm”) can elicit both long-term potentiation and depression. Statistical analysis predicts that the mechanism underlying this bidirectional plasticity involves the proportional addition or removal of presynaptic release sites. Gamma stimulation with the same number of APs as in TBS was less efficient in inducing plasticity. Both TBS- and “single-sniff paradigm”-induced plasticity depend on NMDA receptor activation. Since the onset of plasticity is very rapid and requires little extra activity, we propose that these forms of plasticity might play a role already during an ongoing search for odor sources. Our results imply that components of both short-term and long-term olfactory memory may be encoded at this synapse.

## 1. Introduction

Basal activity of the mammalian olfactory bulb is synchronized to breathing; during odor sensing the principal neurons of the olfactory bulb, the mitral and tufted cells (MTCs), are firing in repetitive bursts that are locked to the breathing rhythm [1–4]. This property is reminiscent of theta-gamma coupling in the hippocampus [5]. In the bulb, the fast component is by now known to be mostly driven by interactions between the excitatory MTCs and the inhibitory granule cells via a special type of microcircuit, a reciprocal synapse between the lateral dendrites of MTCs and the large GC spines that are also known as gemmules. The specific subtype of fast bulbar network oscillation—fast gamma, slow gamma, and/or beta—is related to the principal cell type and/or sublamina of the external plexiform layer involved, as well as the behavioral state of the animal which is reflected in different top-down interactions [6, 7]. MCs and TCs also differ with respect to the respiratory phase that their peaks of activity are locked to and the “burstiness” of their spiking [3, 8, 9].

With respect to a potential site for olfactory memory-related synaptic plasticity in mammals, MTCs project to the piriform cortex and numerous other higher olfactory areas, and subregions of the piriform cortex are involved in the synthesis and categorization of the odor percept, while conscious perception of odors most likely arises from the yet higher orbitofrontal cortex (reviewed in [10]). However, it is known that odor recognition and discrimination, tasks that clearly involve aspects of memory, are to some extent already performed by olfactory bulb circuits, in particular by the reciprocal synapse mentioned above, since modification of GCs' postsynaptic receptors of the NMDA-, AMPA-, and GABA_A_-type was shown to influence the speed of odor discrimination between highly overlapping mixtures and odor learning can be facilitated also by other interventions in GCs [11–14]. Thus, the MTC-GC synapse can decode stimulus properties and might serve as a locus of long-term plasticity.

Theta-gamma coupling in the form of theta burst stimulation (TBS) is known to induce long-term plasticity in the hippocampus, probably even more effectively than the classical high frequency stimulation (reviewed in [15]). As to TBS-induced plasticity at the MTC to GC synapse so far, a study by Ma et al. [16] observed LTD in granule cells following TBS in the external plexiform layer. We used a similar approach, yet based on glomerular stimulation which depolarizes individual glomeruli and thus consistently activates “sister mitral and tufted cells” that belong to the same glomerulus and the surrounding periglomerular circuitry. Compared to extracellular stimulation in the external plexiform layer, this paradigm should correspond to a more physiological situation with respect to activation of bulbar circuits during breathing since it stimulates the sensory input pathway instead of an accidental set of local synaptic connections.

We also applied variations of TBS, in particular a stimulation paradigm that attempts to mimic odor perception during just a single sniff. These investigations might prove illuminating in the context of natural odor sources in the wild because of two aspects. First, due to turbulent airflow odor molecule concentrations are unlikely to decline with distance from the odor source in a monotonic fashion; rather, patches of odor molecules separate from the original odor plume while drifting downwind. Thus odor detection is expected to be highly discontinuous (e.g., [17]), a notion that also increasingly influences studies on insect olfaction [18–21]. Second, it was observed that indeed rats can detect and discriminate odors within single sniffs [22, 23] and that they use casting techniques to track down patchy odor trails [24]. The single-sniff (or rather single-whiff-of-odor) scenario is thus likely to repeatedly occur within tens of seconds during the search for an odor source or during exploration of novel environments.

In summary, our study aims to determine whether plasticity at the MTC-GC synapse can be elicited by induction patterns based on coincident bulbar respiratory rhythmic activity and olfactory inputs.

## 2. Materials and Methods

### 2.1. Preparation of Brain Slices and Whole-Cell Recording

All experiments were carried out according to national and institutional guidelines, the rules laid down by the EC Council Directive (86/89/ECC) and German animal welfare. Animals were deeply anaesthetized with isoflurane and decapitated. Sagittal olfactory bulb brain slices were prepared of juvenile Wistar rats (thickness 300–350 *μ*m; postnatal day (PND) 11–17). Neurons were visualized by infrared gradient-contrast illumination via an IR filter (Hoya, Tokyo, Japan) and patched with pipettes sized 6–8 MΩ. Somatic whole-cell patch-clamp recordings were performed with EPC-9 (HEKA, Lambrecht, Germany). Series resistances measured 10–40 MΩ.

The intracellular solution contained [mM] 130 K-methylsulfate, 10 HEPES, 4 MgCl_2_, 4 Na_2_ATP, 0.4 NaGTP, 10 Na phosphocreatine, and 2 ascorbate, at pH 7.2. The extracellular artificial cerebrospinal fluid was gassed with carbogen and contained [mM] 125 NaCl, 26 NaHCO_3_, 1.25 NaH_2_PO_4_, 20 glucose, 2.5 KCl, 1 MgCl_2_, and 2 CaCl_2_. Due to the fragility of long-term granule cell recordings, experiments were performed at room temperature (~21°C). GCs were identified by their morphological appearance and the shape of current-evoked APs and firing [25]. The average input resistance of the investigated GCs was on the order of 1 GΩ and their resting potential was ranging from −80 to −70 mV, similar to our previous data [25, 26]. Leaky GCs with a holding current above ~−30 pA were rejected. A stable resting potential *V*_*m*_ (within a narrow range of a few mV) was found to be paramount for stable EPSP amplitude recordings; experiments that showed a substantial drift in *V*_*m*_ were rejected.

### 2.2. Extracellular Activation of MTCs

Glomerular stimulation was performed with a custom-built four-channel-electrode (Figure 5(b)). The four electrodes consisted of Teflon-coated silver wires (diameter uncoated 75 *μ*m, coated 140 *μ*m, item AG-3T, Science Products GmbH, Hofheim, Germany) and were aligned in parallel at a distance of 200 *μ*m across two screws with the according pitch and embedded in epoxy glue. The electrode was connected to a 4-channel stimulator (STG 1004, Multi Channel Systems, Reutlingen, Germany), which is controlled from a PC via an USB connection. In current mode, the maximal stimulation strength per channel is 800 *μ*A. The grounds from the stimulator channels were connected to a common wire and then to the bath. Alternatively, the ground was connected to the fourth stimulator wire; however, the first configuration was generally preferred due to its larger choice of stimulation options. The 4-channel electrode was lowered on top of the acute brain slice under visual control using a manual manipulator (Scientifica, East Sussex, UK). The stimulation strength was adjusted via the stimulator's software on the PC; the output of the stimulator was triggered from the electrophysiology software (Pulse, HEKA, Lambrecht, Germany). Stimulation strengths sufficient to elicit GC EPSPs were mostly in the range of 100–400 *μ*A.

The 4-channel electrode was positioned on an olfactory bulb slice such that at least two electrode wires were located within individual glomeruli, usually with one nonstimulated glomerulus in between, since the diameter of a glomerulus is on the order of 100 *μ*m (Figure 5(a)). This arrangement served two purposes, first to increase the success rate for finding connected granule cells and second to test for homosynaptic plasticity via intermittent stimulation of two separate glomerular inputs. Each of the two wires was found to activate independent sets of synaptic inputs onto granule cells, as shown in Figure 5(c).

Previously we have shown that direct glomerular stimulation results in single MC spikes [25], with a latency between stimulation onset and MC AP peak of 4.9 ± 4.4 ms (*n* = 6). The latencies between stimulation onset and the first granule cell EPSP of the responses observed here were thus in accordance with monosynaptic excitation of GCs via MTCs (5.6 ± 2.4 ms, *n* = 28).

### 2.3. Plasticity Measurement Including Induction Protocols

Synaptic plasticity experiments involved

- control: recording of a stable EPSP control at 0.1 Hz for 10 minutes,
- induction: repetition of individual induction protocol at 0.1 Hz, 10 times,
- long-term: recording of EPSPs at 0.1 Hz for at least 30 minutes.

The induction protocols used the same stimulation strength as the control recordings. All phases of the experiments used the same stimulation channel, except for the experiments on homoglomerular plasticity where a second channel was stimulated intermittently and no induction was applied to this second channel. Theta burst stimulation (TBS) involved five bursts at 40 Hz with 4 APs each, spaced at 4 Hz. “Θ-only” stimulation consisted of five APs at 4 Hz and “*γ*-only” stimulation of 20 APs at 40 Hz (same number of spikes as in TBS). Single burst stimulation (SBS) used just one burst at 40 Hz with 4 APs. All induction protocol sequences are shown in Figure 4(a).

### 2.4. Data Analysis and Selection

**Figure 1:**
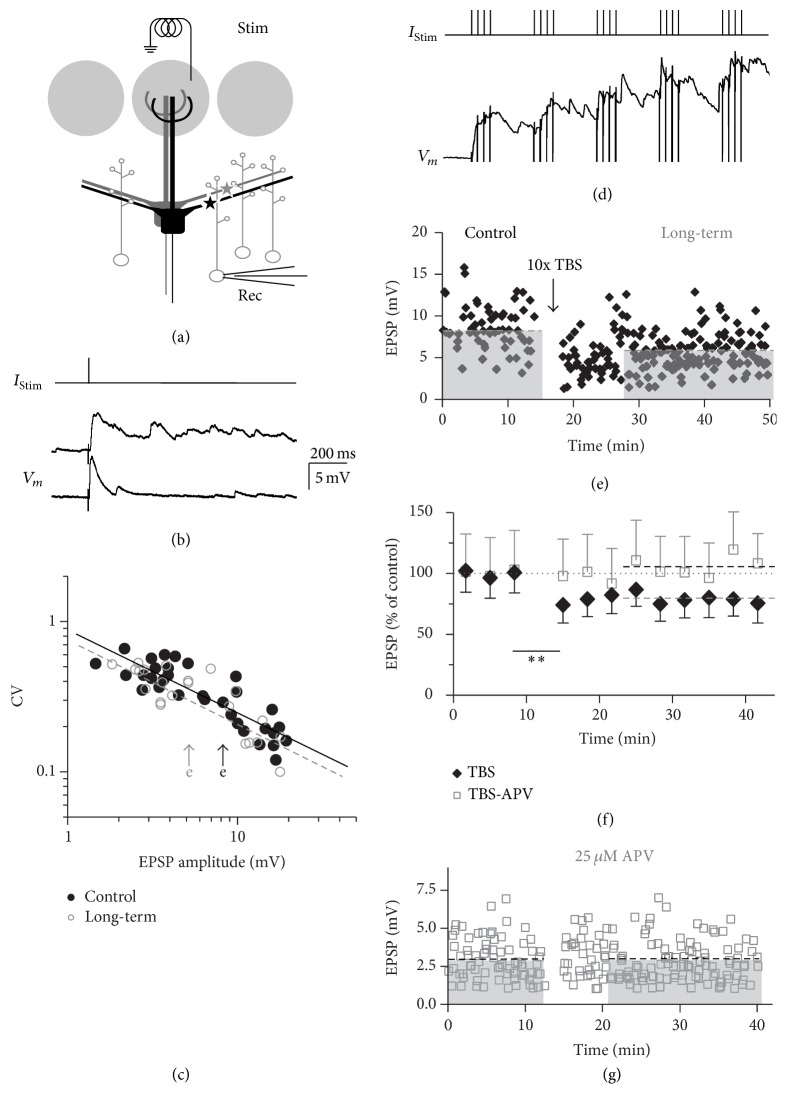
TBS induces LTD at the MTC-GC synapse. (a) Experimental design [25]. Glomerular stimulation (Stim) will activate the glomerular ensemble of mitral cells to a varying extent (depending on stimulation strength); thus inputs from several mitral cells onto the recorded GC (Rec) will be activated. (b) Response of individual granule cell to single glomerular stimulation (two individual responses shown, note the barrage of activity in the second response). (c) Coefficients of variation of EPSP amplitude of synaptic connections versus their mean EPSP amplitude for control data (black dots) and long-term data (grey circles). For control, the fit has a slope of −0.55 ± 0.06 (mean ± SD) on the log scale (black line) and for long-term the slope was −0.56 ± 0.08. The confidence intervals were calculated from *n* = 200 bootstrap replicas of the data. The arrows mark the data points from the connection shown in (e). (d) Response of the cell from (b) to TBS (average of the total of 10 stimulations). (e) EPSP amplitudes from individual GC recordings from the cell in (b) and (d) over time. Control mean 8.1 mV; long-term mean 6.3 mV (dashed lines). (f) Cumulative data (normalized to control) of all experiments with substantial LTD (black diamonds, *n* = 10) and of all experiments in the presence of 25 *μ*M APV (grey squares, *n* = 6). Respective long-term averages shown as dashed lines (grey for TBS and black for TBS in APV). All data points ± SD across experiments. (g) Representative individual experiment in the presence of 25 *μ*M APV. Control mean 2.9 mV; long-term mean 2.7 mV (dashed black lines). ^*∗∗*^The degree of the statistical difference between the last data point before TBS and the first data point after TBS (black diamonds).

Analysis of EPSPs was performed using custom-written software based on Igor Pro (Wavemetrics, Lake Oswego, Oregon), as previously described [26]. Percentage values indicate the change in EPSP amplitude relative to the mean control amplitude. Each data point represents an average of 3.33 min of recording (22 sweeps maximum per point). The average long-term mean EPSP amplitude of an experiment was calculated across the interval of 10–30 min after induction or longer if the recording persisted (see Figure 1(d)). Before averaging across experiments, the EPSP amplitudes of each experiment were normalized to the mean control EPSP amplitude. Failures were very rarely observed and thus not accounted for in the analysis. The coefficient of variation (CV) was calculated as the standard deviation across EPSP amplitudes divided by their mean. It was analyzed only for TBS or SBS experiments; experiments with a high spontaneous activity or multiple response peaks where a precise determination of the first response amplitude was often compromised were excluded from CV analysis.

The criterion for successful induction of long-term plasticity was a stable change in the long-term mean EPSP amplitude of at least ±10% relative to the control average.

Cumulative data of whole sets of experiments are represented as mean ± standard deviation of the mean (SD).

Experimental data points and averages of data sets were compared statistically using nonparametric tests (Wilcoxon test for paired data sets and Mann-Whitney test for unpaired data). All averages are given ± SD unless indicated otherwise.

Short-term plasticity was measured in terms of relative fractions (later EPSP amplitudes divided by first EPSP amplitude). Later amplitudes were measured from the membrane potential right before the respective stimulus artifact, since fitting of the decay of *V*_*m*_ was not possible at 40 Hz. Thus later amplitudes are slightly underestimated, depending on the rise time of the EPSP.

### 2.5. Quantal Analysis of the SBS Experiments

The quantal properties of the synapses measured at the SBS experiments were estimated by fitting the mean and CV of their responses to the relevant measures of the quantal model of synaptic transmission [27]. In particular, a synaptic connection is considered to be composed of *N* independent release sites, from which a maximum of a single vesicle per site is released with probability *p* upon the arrival of a presynaptic action potential. Subsequently, the vesicle contributes a quantum *q* to the postsynaptic response. In the simplest case, in which the synaptic response variability is only governed by the vesicle release events (and not by other noise sources [28]), the expected mean and variance of the responses are

(1) Mean=N·p·q,

(2) Variance=q2·N·p·1−p.

Subsequently, CV is

(3) CVVarianceMean=q2·N·p·1−pN·p·q=1N·1−pp.

We used the following approach in order to fit the quantal parameters *N*, *p*, and *q* to the synaptic connections of the SBS experiments: an average release probability was assumed for the release sites at the control and long-term phases of the experiments, *p*_control_ and *p*_long-term_. For each value of these probabilities (range 0.05–0.95), the number of release sites, *N*_*i*,control_ (or for the long-term phase *N*_*i*,long-term_) that explain best the experimental CV was calculated for each synaptic connection *i* (index *i*, running from 1 to the total number of SBS experiments within the CV analysis). Subsequently, the value of *p*_control_ (*p*_long-term_) and the related set of *N*_*i*,control_ (*N*_*i*,long-term_) that minimized the overall mean-square distance of the fitted CVs from the measured CVs were the values that we assigned to the synaptic connection. Finally, *q*_*i*,control_ (*q*_*i*,long-term_) value of the release sites of a given synaptic connection was calculated from (1), by considering its measured mean response amplitude and the fitted *p*_control_ (*p*_long-term_) and *N*_*i*,control_ (*N*_*i*,long-term_).

## 3. Results

### 3.1. Properties of Synaptic Transmission between Glomeruli and Granule Cells

To elicit granule cell EPSPs we stimulated single glomeruli with a monopolar, four-channel electrode (Figure 1(a); see Section 2.2). Individual MTC to GC synaptic connections are thought to involve no more than one single synaptic contact because of their anatomical arrangement [29], with a median unitary EPSP size at the soma of 2 mV [30]. Since we did not apply minimal stimulation, the recorded EPSPs were mostly not unitary but involved activation of several presynaptic MTCs (average EPSP size across all experiments without presence of APV 8.9 ± 6.4 mV, median 8.3 mV, range 1.4–31.2 mV, *n* = 52; similar EPSP distributions within sets of experiments; see the following).

In roughly half of the experiments, we observed a barrage of network activity following the first EPSP (see Figure 1(b)), which is likely due to stimulation of the OSN axon bundle leading up to a glomerulus, depending on the placement of the four-channel stimulator [31]. For evaluation of plasticity we analyzed only the first EPSP after stimulation; the occurrence of barrages was not correlated with the amplitude of the first EPSP (*r* = −0.05, *n* = 45).

The average coefficient of variation (CV) for these parallel inputs to a single GC was 0.35 ± 0.15 (*n* = 32, excluding experiments where the first EPSP was frequently summated with network activity). Figure 1(c) shows that on the logarithmic scale the CV values were linearly dependent on the mean EPSP amplitude, with fitted slopes of −0.55 ± 0.06 for the control and −0.56 ± 0.08 for the long-term measurement phase (mean ± SD from bootstrapping).

To control for systematic rundown, we performed 5 experiments where we recorded EPSPs for 30 min without any intervention. With a normalized average amplitude in the first 10 minutes (control) of 100 ± 28% (*n* = 177 data points), the average amplitude in the last 10 minutes was 101 ± 29% (*n* = 172 data points). Also, none of the individual experiments showed any depression in EPSP amplitude in the last 10 minutes compared to the first 10 minutes according to our criterion (>10% change below control). Moreover, we did not observe a systematic rundown over the entire recording period (40 min) in experiments with no induction in the recorded glomerular channel (see, e.g., Figure 6(d)). Thus GC EPSPs were generally stable at the basal stimulation frequency of 0.1 Hz.

**Figure 2:**
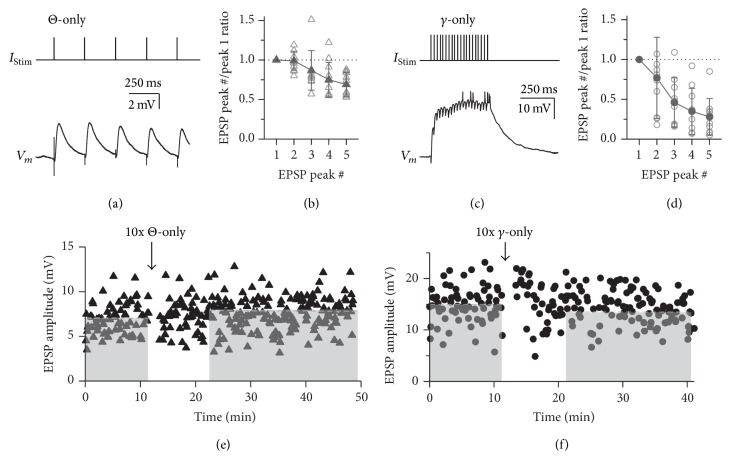
Short- and long-term plasticity in response to Θ-only and *γ*-only stimulation. (a) Representative example of response to Θ-only protocol averaged over the total of 10 stimulations. (b) Cumulative data of EPSP amplitude ratios relative to first peak in train (*n* = 10 cells). Open symbols: individual experiments. Solid symbols: averaged data. (c) Same as in (a) but for *γ*-only protocol. (d) Same as in (b) but for *γ*-only protocol. Cumulative data of EPSP amplitude ratios relative to first peak in train (*n* = 10 cells). (e, f) EPSP amplitudes of individual long-term experiments with induction responses shown in (a) and (c), respectively. Cumulative data for both Θ-only and *γ*-only data sets are shown in Figure 4(a). (e) Θ-only. Control mean 7.5 mV; long-term mean 8.1 mV (dashed lines). (f) *γ*-only. Control mean 15.1 mV; long-term mean 13.0 mV (dashed lines).

At both of the higher stimulation frequencies used in the induction protocols (4 Hz and 40 Hz), the MTC-GC synapses underwent short-term depression after more than two pulses, while paired pulses showed either facilitation or depression, with a prevalence of depression (Figures 2(a)–2(d)). At 4 Hz (*n* = 10 GCs), the mean ratio of the second EPSP amplitude to the first (the paired pulse ratio) was 0.99 ± 0.12 (*P* = 0.15) and from the fifth to the first 0.69 ± 0.15 (*P* < 0.01, cf.  Figure 2(a) for an individual example). At 40 Hz (*n* = 10 GCs), the mean ratio of the second EPSP amplitude to the first was 0.77 ± 0.51 (*P* < 0.05) and from the fifth to the first 0.28 ± 0.23 (*P* < 0.01, cf. Figures 2(b) and 1(d)).

### 3.2. Plasticity Induction by Θ-*γ* Coupling

Our criterion for successful induction of long-term plasticity was a stable change in EPSP amplitude of at least 10% away from the control average that persisted from 10 to 30 min after induction. The TBS protocol reliably induced long-term depression (LTD) of EPSP amplitudes in most GCs tested (to 73 ± 13% of control, *n* = 10 of 14 experiments, *P* < 0.005, Figure 1(d)). If the remaining 4 cells were included, the average depression reached 82 ± 19% of control and was still highly significant (*P* < 0.005, distribution of all TBS-data shown in Figure 4(a)). There was no correlation between the mean control EPSP amplitude and the mean long-term EPSP amplitude (*P* = 0.94).

Onset of TBS-induced LTD was rapid (Figures 1(e) and 1(f)), as also evident from comparing the first data point after induction with control and the later data points: the mean EPSP amplitudes from the first averaged bin after induction were significantly below those from the last bin before induction (comparison of data means across experiments, Wilcoxon test, *P* < 0.01, *n* = 10) and at the same time statistically not different from for example the 5th bins after induction (*P* = 0.45). This observation also argues against systematic rundown.

In *n* = 3 experiments, the stability of GC recordings allowed for more than one induction with TBS. In 2 of these further LTD could be induced at 30 min after the first induction (to 75 ± 9% of the already depressed EPSP). Thus, this form of plasticity is most likely not saturated by a single induction.

Interestingly, postsynaptic spiking during TBS was no prerequisite for LTD induction since EPSP summation sufficient for spike generation occurred in only 7 out of the 14 experiments during induction, including 2 of the 4 experiments with no LTD. The mean long-term values relative to control between the two groups were not significantly different (*n* = 7 each, 87 ± 10% with spikes versus 76 ± 24% without, *P* = 0.18).

Next we tested whether the occurrence of barrages of network activity was related to the degree of plasticity induction. There was no correlation between the half duration *τ*_1/2 of the averaged control compound EPSP and the amount of LTD (*r* = −0.31, *P* = 0.32); changes in network activity after plasticity induction as measured by the ratio of the half duration value of the long-term phase to the control phase were also uncorrelated (*r* = −0.25, *P* = 0.44, each *n* = 12).

NMDA receptors play a major role at the MTC-GC synapse [25, 32]. Therefore, we blocked NMDA receptors by adding 25 *μ*M APV to the bath from the beginning of the experiment. In the presence of APV, TBS no longer resulted in LTD (104 ± 17% of control, *n* = 6, *P* < 0.05 versus TBS without APV, Figures 1(f) and 1(g)).

Next, we were interested to see whether variations of the theta burst pattern might be also effective in plasticity induction. Theta stimulation alone (“Θ-only”; 87 ± 21%, *n* = 8, *P* = 0.11 versus no change, i.e., 100%, including 2 experiments without plasticity induction) or a train of 20 stimulations at gamma frequency (“*γ*-only”; 88 ± 10%, *n* = 9, *P* = 0.07 versus no change, i.e., 100%, including 3 experiments without plasticity induction; equal total number of stimulations in *γ*-only and TBS) were apparently less effective in inducing substantial plastic changes (see Figures 2(a) and 2(d) for individual experiments and Figure 4(a) for cumulative data). However, a more efficient induction of LTD by TBS compared to the two variants of the TBS paradigm could not be proven by statistical analysis due to the large variance across experiments within data sets. The distributions of control EPSP amplitudes for these sets of experiments were statistically indistinguishable from the respective distribution for the TBS experiments (Θ-only versus TBS: *P* = 1 and *γ*-only versus TBS: *P* = 0.49).

### 3.3. Plasticity Induction by “Single Sniff” Stimulation

**Figure 3:**
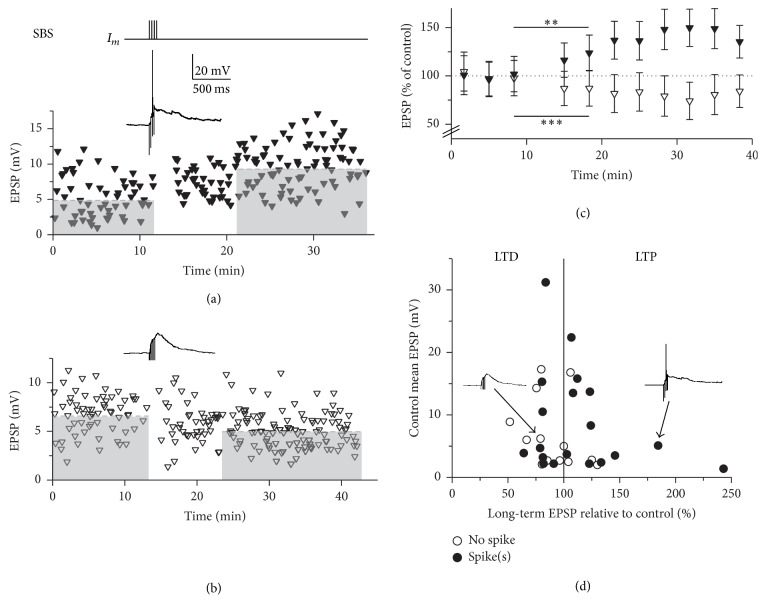
Single burst stimulation (SBS) results in bidirectional plasticity. Top: SBS induction protocol. (a) Individual experiment resulting in LTP. Control mean 5.1 mV; long-term mean 9.4 mV (dashed lines). Inset shows averaged response to SBS stimulation. (b) Individual experiment resulting in LTD. Control mean 6.2 mV; long-term mean 4.9 mV (dashed lines). Inset shows averaged response to SBS stimulation (same scale as in (a)). (c) Cumulative data (normalized to control) of SBS experiments with substantial LTP (solid triangles, *n* = 10) and substantial LTD (open triangles, *n* = 10). All data points ± SD across experiments. From the second data bins after induction onwards, bins were significantly different from the respective last data bins before induction. *∗∗* and *∗∗∗* refer to the statistical comparison between the last data point before induction and the second data point after induction for LTP (black triangles) and LTD (open triangles), respectively. (d) Cumulative display of control mean EPSP amplitudes versus the long-term change of individual experiments normalized to control of individual experiments. Open circles: experiments without action potentials (APs) during SBS; filled circles: experiments with APs during SBS. The two experiments shown in (a, b) are indicated by their SBS averages and arrows.

Since it was observed by several groups that rats and other mammals, including humans, can detect and discriminate odors within single sniffs [33], we have also applied a “single sniff paradigm” using just a single 40 Hz burst for ten times at 0.1 Hz (single burst stimulation, SBS). Subsequently, either LTD or LTP or no plasticity was observed (total experiments *n* = 32; LTD: 76 ± 10% of control, *n* = 13, Figure 3(a); LTP: 145 ± 40%, *n* = 10, Figure 3(b); no effect: *n* = 9). The average long-term mean of all SBS experiments was 104 ± 37%, indicating a possible homeostatic mechanism of plasticity induction (see the following). The distribution of control mean EPSP amplitudes was not significantly different from the respective distribution for TBS experiments (*P* = 0.81).

Again, onset of plasticity was often rapid for both LTP and LTD, as seen in the example experiments in Figure 3. Although overall this change was not yet significant between the last data bin before and the first bin after induction (in contrast to the TBS experiments), it became substantial across experiments for the last bin before and the second bin after induction (3.3–6.7 min after induction) for both LTP (*n* = 10, *P* < 0.01) and LTD (*n* = 12, *P* < 0.005, Figure 3(c)).

Similar to the LTD induced by TBS, bidirectional plasticity was independent of the occurrence of GC sodium spikes (Figure 3(d)) or the maximal depolarization during the induction in experiments without spikes (correlation coefficient *r* = −0.29, *P* = 0.41, and *n* = 18). Larger EPSPs involving more MTC-GC synapses showed less plasticity than small EPSPs (Figure 3(d); see the following).

**Figure 4:**
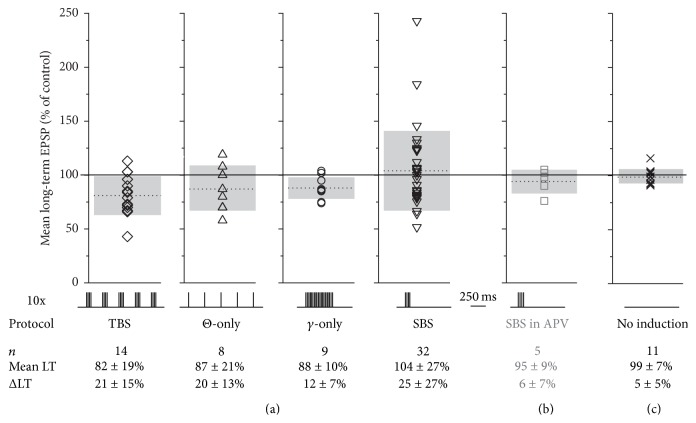
Synopsis of plasticity experiments. SBS plasticity is NMDAR-dependent. Bottom: temporal sequence of induction protocol, number of experiments, average long-term plasticity (mean LT), and average efficiency of long-term plasticity induction (ΔLT, absolute value of plastic changes). All data ± SD. (a) Cumulative display of mean long-term change for all induction protocols with individual data sets, means (dotted lines), and SD (extent of grey bars). (b) Mean long-term change of SBS experiments in the presence of 25 *μ*M APV. Note the substantially reduced variance. (c) Mean long-term change of experiments without induction (*n* = 11: 5 without induction and 6 recorded from heteroglomerular control channel that was not induced with SBS; see Figure 5).

To better compare the effectiveness of the various paradigms for induction of plasticity, we define the parameter “long-term efficiency” or ΔLT as the absolute value of the difference between the average long-term mean relative to control and 100%, that is, control itself, which allows measuring the degree of bidirectional plasticity independently of its sign (ΔLT = |(long term mean normalized to control − 100%)|). The values for ΔLT for all induction paradigms are given in Figure 4(a). Upon statistical comparison of ΔLT across paradigms, only *γ*-only was found to be significantly different from SBS (*P* < 0.05). All paradigms were more efficient than the control experiments without induction (Figure 4(c); versus SBS, TBS: *P* < 0.005; versus Θ-only, *γ*-only: *P* < 0.05).

Since paired pulse ratios (PPR) at an interstimulus interval of 25 ms (40 Hz) were highly variable across experiments (cf.  Figure 2(d)), we tested whether PPRs could predict the direction and/or the efficiency of plasticity induction ΔLT. No significant correlation was found for either direction or efficiency (*n* = 24, *r* = 0.28, *P* = 0.18, and *r* = 0.31, *P* = 0.17).

Next we tested whether the occurrence of barrages of network activity was related to the degree and the sign of bidirectional plasticity induction. There was no correlation between the half duration of the control compound EPSP and the observed plasticity (*r* = 0.20, *P* = 0.29, and *n* = 31). Changes in network activity after plasticity induction as estimated by the ratio of the half duration value of the long-term phase to the control phase were also frequently observed (>20% away from control in 16 out of 31 experiments) but had no net effect across experiments (average 104 ± 28%, *n* = 31) and were also uncorrelated to plasticity (*r* = −0.04, *P* = 0.80, and each *n* = 31). This finding also held, when the analysis was restricted to the subset of experiments with substantial network activity (*τ*_1/2_EPSP_ > 100 ms, *n* = 16). Thus the presence of barrages and changes in their duration were not predictive of bidirectional plasticity and vice versa.

In 4 GCs the recordings were stable enough to allow for a second plasticity induction in a glomerular channel different from the first. In two of the cells, one induction resulted in LTD and the other in LTP; in the two others, one induction did not result in plasticity whereas the other resulted in LTD or LTP. Therefore the occurrence of bidirectional plasticity* per se* is most likely not cell-specific (at least at this age of animals) but rather depends on the summated plastic changes across the activated set of synapses (see also Section 3.5.).

In the presence of 25 *μ*M APV, SBS no longer induced plasticity in any out of 5 experiments (*n* = 5, 95 ± 9%, ΔLT = 6 ± 7%, Figure 4(b); note the strongly reduced variance and ΔLT compared to control SBS, *P* < 0.02). Thus, NMDA receptor mediated signalling is crucial for both TBS- and SBS-induced plastic changes.

### 3.4. Homosynaptic Plasticity

**Figure 5:**
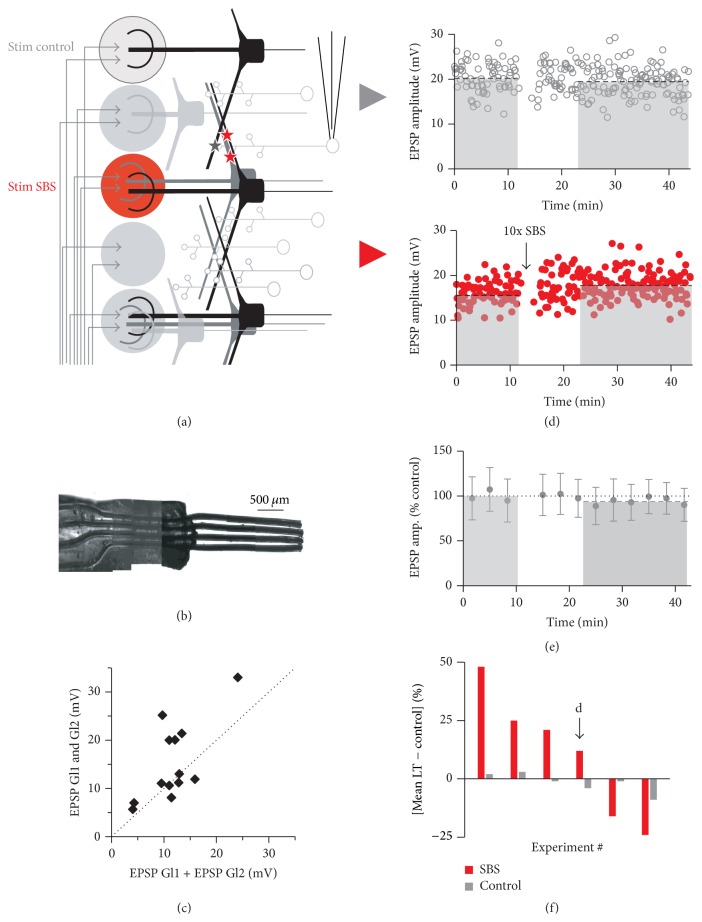
SBS results in homoglomerular plasticity. (a) Scheme of experimental configuration for intermittent stimulation of glomeruli. Two stimulated glomeruli were separated by at least one nonstimulated glomerulus. (b) Photograph of custom-built stimulation electrode (see Section 2). The wires appear wider than their actual size of 75 *μ*m due to their Teflon coating. (c) Test for independence of sets of synapses (*n* = 14 experiments). Scatterplot of EPSP amplitudes of combined activation of two neighboring electrodes versus added EPSP amplitudes in response to individual stimulation of these glomeruli. (d) Individual experiment with intermittent stimulation. Top: control glomerular pathway without induction. Control mean 20.2 mV; long-term mean 19.3 mV (dashed black lines). Bottom: glomerular pathway with SBS induction. Control mean 15.8 mV; long-term mean 17.7 mV (dashed black lines). (e) Cumulative data from control glomerular pathway normalized to baseline (*n* = 6, long-term mean 98 ± 5% of control, dashed grey line). (f) Cumulative display of experiments. Red bars: long-term change (LT mean − control mean) in SBSed glomerular pathway; grey bars: long-term change in control glomerular pathway. The black arrow indicates the experiment shown in (d).

To establish synaptic specificity of SBS-induced plasticity we used the four-channel stimulation electrode for the independent activation of two glomerular inputs on a given GC. To prevent coactivation by extracellular currents, stimulated glomeruli were separated by at least one nonstimulated glomerulus (see Section 2; Figures 5(a) and 5(b)). EPSP amplitudes from two distinct glomerular pathways did sum linearly or supralinearly upon combined stimulation of both pathways, indicating nonoverlapping sets of activated synapses (*n* = 13 GCs, ratio sum individual stimulations to combination 1.35 ± 0.53, *P* < 0.05 compared to strictly linear summation, Figure 5(c)). The supralinearity occurred mostly for larger input amplitudes >10 mV (sum of both EPSPs) and might be due to activation of GC T-type Ca^2+^ channels [30] or other dendritic voltage-dependent mechanisms such as NMDA receptors (see, e.g., [34]).

For plasticity induction, only one glomerulus was stimulated with the SBS protocol while the other served as control input pathway. Figures 5(d)–5(f) show that the observed plasticity was specific to the input pathway which received the plasticity induction protocol, since it was not registered in the neighboring glomerular control input pathways (*n* = 6, total induced plasticity in stimulated input pathway ΔLT = 24 ± 14%, total change in control pathways ΔLT = 3 ± 3%, *P* < 0.025; independence of inputs tested for all these experiments as described above). Thus, this type of plasticity is clearly “homoglomerular,” a finding that most likely also holds for TBS-induced LTD (not tested).

### 3.5. Mechanism of the SBS-Induced Plasticity: Changes in the Number of Release Sites

**Figure 6:**
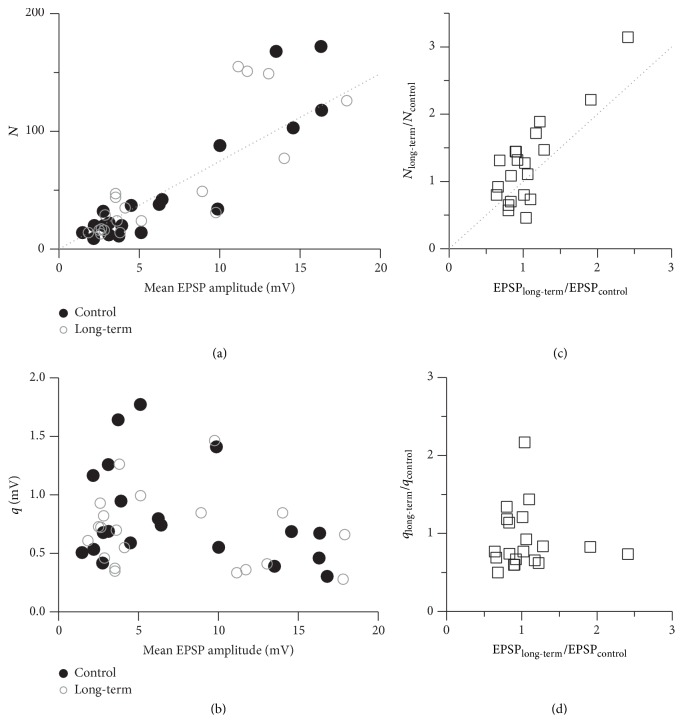
Plasticity induction relies on changes in *N*. Population quantal analysis of the synaptic connections from the SBS experiments. (a) The numbers of release sites *N* were strongly correlated with the synaptic efficacy at both the control (*r* = 0.91, *P* < 0.01) and long-term (*r* = 0.82, *P* < 0.01) measurement phases. The dotted line represents the linear relation between *N* and EPSP amplitude (see (1) in Section 2) for the averaged quantal size 〈*q*〉 = (*q*_control_ + *q*_long-term_)/2 = 0.80 mV and the averaged release probability 〈*p*〉 = (*p*_control_ + *p*_long-term_)/2 = 0.265 resulting from the quantal analysis: *N* = (Mean  EPSP)/(〈*p*〉〈*q*〉). (c) Scatterplot of the relative change in the number of release sites *N* versus the observed relative plasticity. There is a strong correlation (*r* = 0.83, *P* < 0.01). (b, d) In contrast, the quantal sizes *q* and the relative change in their sizes were not correlated with the synaptic efficacies and the observed relative plasticity.

The observed slopes of −0.5 of the linear relations between the CV and the mean of the synaptic responses (in the logarithmic space, Figure 1(c)) indicate a specific mechanistic explanation for the bidirectional plasticity. That is, when a synapse becomes stronger (or weaker), it is mainly due to an increase (decrease) in its number of release sites and not due to changes in the release probability or quantal size [35, 36]. To examine the extent to which this mechanism can explain the plasticity at the MTC to GC synaptic connections, we performed a simple quantal analysis of the synaptic connections for which the CV could be determined at both the control and long-term phases of the SBS experiments (*n* = 20). We have found that the number of release sites at the different synaptic connections was indeed strongly correlated with their efficacy in both experimental phases (control: *r* = 0.91, *P* < 0.001; long-term: *r* = 0.82, *P* < 0.001; Figure 6(a)). In particular, the changes in the number of release sites were strongly correlated with the plasticity in the synaptic efficacies, that is, the mean EPSP amplitude (*r* = 0.83, *P* < 0.001; Figure 6(c)). The average release probability of the connections, on the other hand, was similar before and after plasticity was induced, with *p*_control_ = 0.25 ± 0.05 and *p*_long-term_ = 0.28 ± 0.07 (mean ± SD; calculated from 200 bootstrap replicas of the data). The calculated quantal sizes, with a mean and standard deviation of *q*_control_ = 0.82 ± 0.41 mV and *q*_long-term_ = 0.78 ± 0.35 mV, were not correlated with the synaptic efficacy (Figure 6(b)). That is, the release sites of both weaker and stronger synaptic connections had comparable quantal sizes, and the quantal size and release probability of new release sites, which were added at potentiated connections, were of the same magnitude as for the existing release sites. Similarly, the release sites that disappeared at depressed synaptic connections had the same average size and release probability as the remaining sites (Figure 6(d)).

## 4. Discussion

### 4.1. Functional Role of Long-Term Plasticity Induced by Bursting Activity in the Sensory Input to GCs

We have shown that physiologically motivated induction protocols are capable of inducing long-term plasticity at the MTC-GC synapse. In line with the experiments by Ma et al. [16] who used extracellular stimulation in the external plexiform layer, we find that TBS of MTC inputs via glomerular stimulation results in LTD to about 80% of control. Both Ma et al. [16] and Gao and Strowbridge [37] observed LTP following TBS or spike-timing dependent plasticity (STDP) protocols of cortical inputs onto GCs but did not show LTP at the MTC-GC inputs. We now provide a proof of principle for LTP at this synapse, using a “single-sniff” paradigm. These findings—of both LTD and LTP—are conceptually relevant to prove that these synapses could actually participate in olfactory memory formation (e.g., [38]). In particular, we show that LTP requires short bursts of activity, since longer trains at 40 Hz (*γ*-only) were not effective for inducing LTP.

In olfaction, short bursts should correspond to physiologically relevant sensory inputs, because of both respiratory patterning and the properties of olfactory stimuli in the wild (see Section 1). In a sense, there appears to be a plastic resonance at short bursts compared to longer input at the MTC-GC synapse. Taking into account also the rapid induction of both TBS-LTD and SBS plasticity, it is tempting to speculate that these forms of plasticity might facilitate the search for distant odor sources.

### 4.2. Mechanism and Function of Bidirectional Plasticity

Bidirectional plasticity as observed here for the “single-sniff” paradigm is also known from a few other synapses. In the olfactory bulb, Pimentel and Margrie [39] observed local excitatory glomerular interactions between mitral cell apical dendritic tufts that were mediated by AMPA receptors and that underwent bidirectional plastic changes in response to TBS. Since we did not observe bidirectional plasticity for TBS, it is unlikely that our glomerular stimulation technique also acted at the same site, rather than at the MTC-GC synapse. Thus, the olfactory pathway seems to dispose of several loci for bidirectional tuning. Other examples for bidirectional plasticity were observed in the lateral amygdala [40], following pairing of synaptic stimulation and dendritic Ca^2+^ spikes. Since in our experiments bidirectional plasticity occurred preferentially for smaller inputs, an involvement of Ca^2+^ spikes is unlikely even though these spikes exist also in GCs [25, 41]. Similarly, we found that global GC Na^+^ spikes also had no apparent influence on bidirectional plasticity induction in GCs (see also Section 4.4). Therefore, STDP is unlikely to play a role at GC reciprocal spines. Although bidirectional modifications based on STDP have been reported at a huge diversity of synapses [42], including the olfactory nerve to mitral cell synapse where short bursts were paired with EPSPs [16], STDP is fundamentally different from the plasticity described here since in STDP the direction of plastic change depends on the relative timing between the post- and presynaptic activity and thus can be tuned experimentally.

As to the mechanism of bidirectional plasticity, we have found that both before and after plasticity induction the relation between the CV of the synaptic responses and their mean amplitude is linear with a slope of −0.5 (on the logarithmic scale). Together with the results of our basic quantal analysis this observation implies that the bidirectional changes in synaptic strength due to the SBS stimulation protocol can be explained by the addition or removal of release sites (rather than changes in quantal size or release probability), similar to previous findings for cortical L5 glutamatergic connections [35, 36]. The release probability that we found was lower than at the cortical connections (~0.2 versus ~0.45). Since optical quantal analysis yielded an average release probability of 0.5 at a given reciprocal spine [25] and there are certainly less dendrodendritic synaptic contacts between a glomerulus and GC than the number of release sites obtained from the quantal analysis (Figure 6(a); [43]), a synaptic contact is predicted to contain on average 3 release sites ((1 − (1 − *p*_*r*_)^3^) = 0.49, neglecting potential axodendritic inputs). This prediction is further supported by the quantal size obtained here (~0.75 mV), because the median unitary EPSP amplitude at this connection was found to be ~2 mV [unpublished observations, [30]]. The rather large value of *q* in comparison to the cortical connections (~0.1 mV) could be explained by the compactness of the GC dendritic tree and the hybrid nature of this axodendrite that involves local postsynaptic amplification of EPSPs via various mechanisms [44].

Notably, at cortical synapses LTP is thought to be initially governed by modifications to the release probability, while the structural changes follow on a longer time scale [45, 46]. At the MTC-GC synapse, on the other hand, it appears as if the structural changes are rapid, suggesting an adapted mechanism for fast and stable structural modifications even for short stimuli such as the SBS.

Bidirectional plasticity as observed here is homeostatic, thus the total GC excitability would not be changed via individual short odor samplings. While larger data sets are required to fully establish this important observation, the SBS stimulation type might lead to an exploration within the neural circuit, in search for synaptic pathways that would react with a meaningful response. In reinforcement learning theory such a characteristic follows from the lack of a correlation between stimuli and the reward to the system (i.e., constant reward) [47, 48] and the observed changes may therefore depend on a random state of a yet unknown component of the synapse. Since the experiments were done in acute slices, the observed plasticity is independent of external reward top-down signals. However, in an intact brain this plasticity might be directed via centrifugal inputs on GCs [49].

Although SBS on the whole was found to act homeostatically, recurrent inhibition and lateral inhibition will be tuned at individual contacts between MTCs and GCs, such that different MTCs will be differentially modulated. Thus, bidirectional plasticity might be relevant for the establishment and refinement of the highly complex receptive fields GCs have been suggested to dispose of. The precise nature of these receptive fields is as of yet not fully known but might be related for example to discontinuous representations of the stimulus within the bulb [50], to an incomplete inhibitory mirror image of odorants provided by GCs and governed by centrifugal inputs [51], to the formation of GC columns corresponding to glomerular functional units [52], or to dynamic connectivity as invoked by activity-dependent lateral inhibition [53]. Bidirectional plasticity might also subserve the optimization of the complex and sensitive temporal coding in the bulb, such as fast correlation and slow decorrelation in heteroglomerular simultaneous MC spike trains [54] or the latency coding powered specifically by mitral cells [1, 3]. Finally, MCs were recently reported to undergo a decrease in activity in relation to olfactory memory formation* in vivo* [55], which might require the form of LTP at the MC-GC synapses described here.

While at the network level bidirectional plasticity would affect mainly local processing at individual GC spines, TBS-induced LTD will reduce GC overall excitability within a rather short time span and thus reduce the amount of global lateral inhibition provided by the GCs associated with the active glomerulus. Thus its influence will be downregulated, with further interesting implications for bulbar network processing. Whether local recurrent inhibition is also reduced depends on the precise mechanism of TBS-LTD; if LTD was ultimately related to an increase in spine neck resistance (as suggested above for SBS-mediated plasticity), local recurrent inhibition could be even increased. A stimulation paradigm capable of inducing mainly LTP still remains to be discovered, since LTP would be required to implement, for example, a sparsification of mitral cell responses to known odors [56] or other aspects of olfactory memory. Since reliable correlations between glomerular network activity and the plasticity of input to GCs could not be observed within the scope of our study, interactions between MC-GC plasticity and glomerular processing remain to be elucidated.

### 4.3. Sources of Variability That Might Also Contribute to Bidirectional Plasticity

Several other factors might also play a role in the phenomenon of bidirectional plasticity. First, the short-term plasticity of the MTC-GC synapse described here is in line with previous voltage clamp experiments [54, 57, 58] where both depression and facilitation were observed for paired pulse inputs onto GCs. Interestingly, facilitation was found to be a property of proximal inputs on the GC apical dendrite (which are thought to consist of both cortical inputs and mitral cell axon collaterals), whereas inputs from reciprocal spines rather underwent depression [41, 59]. Since the cortical feedback loop was most likely not conserved in our slice preparation, the facilitating cases might correspond to sets of synapses that consisted predominantly of proximal inputs established by MC axon collaterals, whose influence on GC processing is not well known. This diversity of inputs could also contribute to bidirectional plasticity; however, we did not observe any correlation between short-term and long-term plasticity.

Another source of variability might originate from glomerular activation of disynaptic pathways onto GCs, in particular via inhibitory deep short-axon cells [31, 60], which might also undergo plastic changes.

Similarly, even though we found that bidirectional plasticity was most likely not specific to a given cell, bidirectional plasticity might be due to differing maturational stages of GCs and their synapses, since the early development of the olfactory bulb network is not yet terminated in two-week old animals (e.g., [61]). While the GCs in our sample appeared mature with respect to their anatomy and action potential firing [62], more subtle gradations of maturation could affect plasticity. Also, at this age rodents just begin to actively explore their environment and sniffing behavior is about fully established around PND 11 [63, 64]. Moreover, adult-born GCs differ in their plasticity from early-born GCs, as documented for an NMDAR-independent form of TBS-LTP in new, adult-born GCs [42]. A recent* in vivo* study by Alonso et al. [65] showed that optogenetic stimulation of specifically adult-born GCs (versus early-born GCs) during an olfactory memory task could facilitate learning when applied at 40 Hz (with exactly the same duration as in our “*γ*-only” experiments) rather than at 10 Hz. Since our GC population consists entirely of early-born GCs, it remains to be tested whether the observed weak “*γ*-only” plasticity would be enhanced in adult-born GCs in acute slices.

Finally there are several subtypes of both MTCs and GCs [66] which also might differ with respect to their synaptic plasticity.

### 4.4. Mechanisms of Long-Term Plasticity: Role of the NMDA Receptor in GCs

Blockade of NMDA receptors abolished both TBS-induced LTD and bidirectional plasticity. So, as in many other established forms of long-term plasticity, the NMDA receptor appears to be the key element mediating plastic effects. In contrast to one of the main dogmas held with respect to NMDA receptor mediated long-term plasticity, the occurrence of spikes and the total maximal depolarization during induction as measured at the GC soma were not correlated to the outcome of plasticity induction for both paradigms.

At the MTC-GC synapse, NMDA receptors are already involved in normal synaptic transmission and postsynaptic Ca^2+^ signalling [25, 67], even though the resting potential of GCs is rather hyperpolarized. This observation can now be explained by the strong local postsynaptic depolarization within GC reciprocal spines that is powered mostly by AMPA receptors; NMDA receptor activation appears to occur independently of additional local Na_v_-channel mediated boosting [44]. Thus, plasticity induction is probably based on purely local signalling at the GC spines (although a coupling to regional dendritic events or additional contributions by non-GC NMDA receptors located, e.g., within the stimulated glomerulus cannot be excluded from our data).

Therefore the contribution of the NMDA receptor to plasticity induction must rely on a cooperative effect that occurs during burst stimulation, for example, a summation of postsynaptic Ca^2+^ levels past a certain threshold, while independent of backpropagation of action potentials or dendritic spikes. Such a scenario might also explain bidirectional plasticity by involving two site-specific thresholds that feed into opposing plasticity mechanisms (e.g., [68]).

If cooperative entry of Ca^2+^ is indeed required for plasticity induction, a remaining conundrum is why high frequency stimulation with 20 spikes at 40 Hz (“*γ*-only”) was not effective. Why and how is Ca^2+^ rise cancelled in this case? Larson and Munkácsy [15] similarly found that TBS was more efficient in plasticity induction in the hippocampus than high frequency stimulation (HFS) with the same number of spikes; they propose an underlying depletion of glutamate during HFS. However, this scenario still does not explain the higher induction efficiency of SBS compared to “*γ*-only.” Rather, there might be a negative feedback signal triggered by an “overstimulation” with glutamate and/or postsynaptic Ca^2+^, for example, via MTC or GC mGluRs or the TRPC channels in GC spines that require strong, NMDA receptor dependent stimulation to become activated [69, 70]. Finally, “*γ*-only” might also trigger local plastic changes that are not seen at the GC soma because of filtering by the reciprocal spine neck resistance *R*_neck_ [44], changes that nevertheless could affect GABA release from the spine, for example, via modulation of high-voltage-activated Ca^2+^ currents. Moreover, changes in *R*_neck_ might provide a general explanation for the fast plasticity induction at the MTC-GC synapse, similar to the rapid *R*_neck_ decrease associated with LTP that has been observed for hippocampal spines [59].

### 4.5. Sniffing and Olfactory Coding; Theta-Gamma Coupling in the Olfactory Bulb and in the Hippocampus

Ongoing theta in the hippocampus is reported mostly from rats and mice (but not bats and humans [71]) and is most likely driven from extrahippocampal structures such as the medial septum [72]. Ongoing theta in the olfactory bulb on the other hand appears to be a universal phenomenon in breathing vertebrates, with both MTC and GC membrane potentials being modulated by it (see Section 1 and [73–75]). Both theta and fast oscillations are most likely originating within the bulb itself [76, 77], with the fast oscillations being generated at the MTC-GC reciprocal synapse [6, 7]. Hippocampal and bulbar theta rhythms are reciprocally transmitted to the bulb and hippocampus, respectively [72, 78–80].

While gamma oscillations coordinate activity of neurons on the time scale of EPSPs and thus would be well suited to provide patterns of activity that are optimal for the induction of STDP, we did not observe a prominent role of spikes in GC plasticity. In analogy to observations in the hippocampal system, the observed plasticity at the MTC-GC synapse might not just be induced by TBS but might also be acting as a driver for gamma bursting via recurrent inhibition, since nested gamma bursts in the entorhinal cortex were shown to be driven by TBS via feedback inhibition [81]. Also, gamma oscillations could serve to feed into theta via dendritic integration in both mitral and granule cells involving slow conductances such as HCN and TRP channels, as proposed for hippocampal CA1 neurons [82]. Finally, the higher frequency sniffing of rodents during active exploration enhances gamma power [83], which might also contribute to a higher degree of plasticity. Interestingly, active sampling of odor plumes in insects was also found to synchronize oscillations in antennal lobe networks, in line with other plastic changes on a short time scale [20].

## 5. Conclusions

In summary, we have demonstrated burst-induced forms of plasticity of the MTC-GC reciprocal synapse with a fast onset that might endow the olfactory system with the sensitivity required for fast learning of new, weak stimuli, for example during exploration. This property might be particularly relevant for olfaction because of the huge space of potential stimuli most of which is unknown to the organism at any point in its life. The finding of both LTD and LTP at this synapse is relevant for its versatility within bulbar processing.

## Abbreviations

AP: Action potential
GC: Granule cell
MTC: Mitral and/or tufted cell
TBS: Theta burst stimulation
SBS: Single burst stimulation
PND: Postnatal day
LTP: Long-term potentiation
LTD: Long-term depression
CV: Coefficient of variance.
